# A Comparison of 18-Gauge and 20-Gauge Short Peripheral Catheters in Terms of Pain and Procedural Difficulty: A Randomized Controlled Trial in the Emergency Department

**DOI:** 10.3390/medicina62091747

**Published:** 2026-09-10

**Authors:** Erhan Altunbas, Emir Unal, Onatcan Ozogul, Cigdem Ozpolat, Sinan Karacabey

**Affiliations:** 1 Department of Emergency Medicine, Marmara University School of Medicine, 34854 Istanbul, Türkiye; 2 Department of Emergency Medicine, Marmara University Pendik Training and Research Hospital, 34899 Istanbul, Türkiye

**Keywords:** short peripheral catheter, peripheral intravenous catheterization, catheter gauge, pain, emergency department, randomized controlled trial

## Abstract

*Background and Objectives*: Clinicians often default to the narrower short peripheral catheter (SPC), believing wider catheters to be more painful and harder to insert. One previous randomized trial found no difference, but it neither selected patients by measured access risk nor interpreted the result against a prespecified threshold of clinical importance. We conducted a confirmatory trial addressing both, comparing 18-gauge and 20-gauge SPCs in adult emergency department patients at low-to-moderate risk of difficult access. *Materials and Methods*: In a single-center, parallel-group superiority randomized controlled trial with 1:1 allocation (ClinicalTrials.gov NCT07629427), adults with an Adult Difficult Intravenous Access (A-DIVA) score from 0 to 3 were allocated to an 18-gauge or a 20-gauge SPC by block randomization. Patients were blinded by goggles until dressing, and the data analyst was also blinded. Co-primary outcomes were patient-reported pain and nurse-reported procedural difficulty on 100 mm visual analog scales immediately after the first attempt; first-attempt success was secondary. The trial was powered for a 13 mm difference in pain. *Results*: In total, 204 patients were randomized, 102 per group. Median pain was 32.5 vs. 30.5 mm (median difference 1 mm, 95% CI −4 to 6; *p* = 0.65) and median procedural difficulty was 12.0 vs. 15.0 mm (−1 mm, 95% CI −4 to 1; *p* = 0.28). The first attempt succeeded in 92.2% and 90.2% of patients (absolute risk difference 2.0 percentage points, 95% CI −5.8 to 9.7; *p* = 0.62); no prespecified subgroup showed a treatment interaction. *Conclusions*: In adults at low-to-moderate risk of difficult intravenous access, we did not detect a difference between 18-gauge and 20-gauge SPCs in terms of insertion pain, procedural difficulty, or first-attempt success, and the confidence interval for pain lay well within the minimum clinically important difference. Formal equivalence was not tested; the trial was designed to detect superiority. SPC size can therefore be chosen on the anticipated therapy rather than the expected discomfort.

## 1. Introduction

The insertion of a short peripheral catheter (SPC)—the device also referred to as a peripheral intravenous catheter or cannula [1]—is one of the most frequently performed invasive procedures in medical and nursing practice, required for blood sampling, fluid resuscitation, the administration of medications and blood products, and contrast-enhanced imaging [2,3,4]. It is particularly important in the emergency department (ED), where patients may deteriorate rapidly and treatment is often time critical. First-attempt success in adults is nonetheless variable, with reported rates ranging from 73% to 86% [5,6], leaving a substantial minority of patients who undergo repeated attempts before access is obtained.

The choice of SPC size involves competing considerations. A wider SPC permits the faster delivery of fluid and contrast, which matters when rapid volume resuscitation may be required, although the gain is smaller than the Poiseuille relationship predicts because flow through a SPC is not fully laminar at the rates used clinically [7]. This advantage is offset by a widely held belief that wider SPCs are more painful and more difficult to place, which leads clinicians to select the smaller SPC by default and may leave the patient with inadequate access if the condition deteriorates [8]. Cannulation is uncomfortable and often anxiety-provoking whatever the size, and each failed attempt adds to the patient’s pain, delays in treatment, and dissatisfaction with care [9,10,11]. The available evidence, however, suggests that insertion pain depends less on the width of the SPC than on whether the attempt succeeds, together with patient-level factors such as sex, puncture site, and the underlying difficulty of access [10]; the discomfort and difficulty commonly attributed to larger SPCs may therefore reflect failed attempts rather than SPC size.

Difficult intravenous access is most often defined as failure of the first cannulation attempt, the outcome around which risk-prediction tools have been built [2]. The Adult Difficult Intravenous Access (A-DIVA) scale, developed and subsequently validated across multiple centers, identifies these patients beforehand from a small set of bedside observations, assigning them to low-, moderate-, or high-risk categories across which the probability of first-attempt failure increases steadily [2,12]. Because access difficulty strongly influences the number of attempts, the pain experienced, and the likelihood of success, any comparison of SPC sizes must take it into account, most simply by restricting enrollment to patients at low-to-moderate A-DIVA risk.

Studies providing direct comparative evidence regarding SPC size are limited. In a cross-sectional study of 1063 surgical patients, SPC size showed no independent association with insertion pain once sex, American Society of Anesthesiologists (ASA) classification, A-DIVA risk, puncture site, and the success of the attempt were taken into account [10]; the ‘’Success and Pain associated with Intravenous Cannulation in the Emergency Department’’ (SPICED) trial randomized 178 emergency patients to an 18-gauge or a 20-gauge SPC and found no difference in either of its co-primary outcomes, i.e., patient-reported pain and clinician-reported procedural difficulty [8]. Neither study used a validated instrument for difficult access to define eligibility, so both pooled patients across the full range of access risk, and any effect of SPC size could have been diluted or exaggerated by case mix; neither expressed the treatment effect against a threshold of clinical importance defined in advance, so a null result cannot readily be distinguished from an underpowered one. Whether that conclusion holds in a population selected by measured access risk, and against a difference defined in advance as clinically important, therefore remains an open question.

We conducted a single-center, parallel-group, randomized controlled trial comparing 18-gauge and 20-gauge SPCs in adult ED patients at low-to-moderate A-DIVA risk. The trial was conceived as a confirmatory replication of the SPICED trial rather than as a first examination of the question. It differs from that trial in restricting eligibility by a validated instrument for difficult access, in judging the observed difference against a minimum clinically important difference specified in advance, in a larger sample sized on that margin, and in prespecified subgroup analyses of first-attempt success. The co-primary outcomes were patient-reported pain intensity and nurse-reported procedural difficulty, each measured immediately after the first cannulation attempt; first-attempt cannulation success, overall and by cannulation site; operator experience; A-DIVA risk category; age; and sex were the secondary outcomes. The trial was designed as a superiority trial and tested the null hypothesis of no difference between the two SPC sizes in insertion pain and in procedural difficulty. It was sized to detect a difference of 13 mm in pain intensity, the accepted minimum clinically important difference on a 100 mm visual analog scale; procedural difficulty and first-attempt success were not separately powered. We anticipated that any difference between the two sizes would be smaller than the minimum clinically important difference, but the trial was not designed as an equivalence study.

## 2. Materials and Methods

### 2.1. Study Design and Setting

We conducted a single-center, prospective, parallel-group superiority randomized controlled trial with a 1:1 allocation and blinding of patients and the data analyst in the adult ED of Marmara University Pendik Training and Research Hospital, Istanbul, Türkiye, in July 2026. Reporting follows the Consolidated Standards of Reporting Trials (CONSORT) statement [13]. The study was approved by the Marmara University Faculty of Medicine Clinical Research Ethics Committee (approval no. 09.2026.774) and was prospectively registered at ClinicalTrials.gov (NCT07629427) before the first participant was enrolled. Before enrollment, written informed consent was obtained from every participant or, where appropriate, from a relative or legal guardian.

### 2.2. Participants

Adults aged 18 years or older who required peripheral intravenous access for diagnostic or therapeutic reasons were eligible and were enrolled consecutively. So that the effect of SPC size could be examined without the confounding weight of difficult access, we limited enrollment to patients at low-to-moderate risk on the A-DIVA scale, defined as a score of 0 to 3 [2,12]. The scale draws on a small set of bedside observations to place patients in low- (0–1), moderate- (2–3), or high-risk (4–5) categories, and the chance of a failed first attempt climbs steadily across them [2,12]. The score was assigned by the nurse who went on to perform the cannulation.

Patients were excluded if they could not reliably report pain intensity—owing to cognitive impairment, altered mental status, or a visual or language barrier—or if they were hemodynamically unstable, defined as a systolic blood pressure below 90 mmHg or a mean arterial pressure below 65 mmHg.

### 2.3. Randomization, Allocation Concealment, and Blinding

Eligible, consenting patients were allocated 1:1 to the 18-gauge or the 20-gauge group from a computer-generated block randomization sequence with a fixed block size of four. A statistician not otherwise involved in the trial generated the sequence, participants were enrolled by investigators, and the attending nurse implemented the allocation by opening the envelope. Assignments were concealed in sequentially numbered, sealed, opaque envelopes, each opened only once a patient had been enrolled and had consented; the sequential numbering and opaque envelopes were intended to keep upcoming allocations unpredictable despite the fixed block size.

Patients wore blackout goggles from before the start of the procedure until the insertion site had been covered with an opaque dressing; the goggles were then removed and the patient marked the pain score, so that the patient remained unaware of the SPC size at the time of scoring. The SPCs are color-coded (18-gauge, green; 20-gauge, pink), so the inserting nurses could not be blinded, nor could the observer who was present during the procedure and administered the pain scale since the SPC was visible at the bedside. Procedural difficulty was necessarily reported by the unblinded operator. The statistician who analyzed the data was a different person from the one who generated the allocation sequence and was blinded to group assignment.

### 2.4. Interventions

In both arms, ED nurses performed cannulation under standard antiseptic and securement procedures, targeting the dorsum of the hand, the forearm, or the antecubital fossa, and no local anesthetic was used. The nurse on duty at the time of enrollment performed the cannulation; all nurses working in the department were eligible to act as operators regardless of their length of experience, and no additional training was provided for the trial. The insertion site and the target vein were selected by the operator after the allocation envelope had been opened. Patients in the 18-gauge arm received an 18-gauge (green) SPC and those in the 20-gauge arm received a 20-gauge (pink) SPC (Hi-Tech Medics Private Limited, Gorakhpur-Uttar Pradesh, India). When a first attempt failed, at least one further attempt with the assigned SPC was required before the operator was free to choose the SPC size and site for any subsequent attempt.

### 2.5. Outcomes

The co-primary outcomes were patient-reported pain intensity and nurse-reported procedural difficulty, both recorded immediately after the first cannulation attempt and both registered as primary outcomes. Pain was marked by the patient on a 100 mm visual analog scale (VAS) from 0 (no pain) to 100 (worst imaginable pain) under the blinding conditions described above. Procedural difficulty was rated by the operator on a separate 100 mm VAS from 0 (no difficulty) to 100 (worst imaginable difficulty). The sample size was based on pain intensity, for which a minimum clinically important difference has been established; no such difference has been defined for a procedural difficulty scale, and the trial was therefore not separately powered for that outcome.

The secondary outcome was first-attempt cannulation success, defined as placement achieved on the first skin puncture and confirmed by entry into the vein with flashback of blood. It was compared between groups overall and across subgroups defined by cannulation site, operator experience, A-DIVA risk category, patient age, and patient sex. Operator experience was recorded as months of clinical experience and, for subgroup analysis, grouped as ≤12, 13–60, or >60 months; age was grouped as under and over 65 years. These subgroup comparisons were prespecified and registered but remain exploratory, as the trial was not powered to detect effects within subgroups. The total number of attempts required to obtain access was also recorded and is reported descriptively.

### 2.6. Sample Size

The sample size rested on the primary outcome, pain intensity. Taking a minimum clinically important difference of 13 mm [14,15,16], a standard deviation of 27 mm (2.7 on the 0–10 scale) from earlier studies of cannulation pain [8], a two-sided alpha of 0.05, and 90% power, 92 patients were needed per group. After allowing for 10% attrition, the enrollment target was 102 patients per group, or 204 in total.

### 2.7. Statistical Analysis

Analyses followed the intention-to-treat principle, with patients kept in the groups to which they were randomized; a per-protocol analysis restricted to patients who received the assigned SPC as specified was run as a sensitivity check. Both co-primary outcomes were compared with the independent-samples *t*-test, or the Mann–Whitney U test where the Shapiro–Wilk test indicated non-normality, and are summarized as mean (standard deviation) or median (interquartile range) accordingly. The between-group effect for each is given as the Hodges–Lehmann median difference (18-gauge minus 20-gauge) with its 95% confidence interval. This estimator is the median of all between-group pairwise differences and need not equal the difference between the two sample medians. First-attempt success was compared with the chi-square test, or Fisher’s exact test where expected counts were small, and is reported as an absolute risk difference and as an odds ratio, each with a 95% confidence interval; the prespecified subgroups were examined through treatment-by-subgroup interaction terms in logistic regression.

Two analyses were post hoc and are labeled as such throughout: the subgroup estimates of pain and procedural difficulty, and a multivariable logistic model of first-attempt success containing SPC size, A-DIVA category, and operator experience. With only 18 first-attempt failures, the model was limited to these three variables, giving 4.5 events per coefficient, and maximum-likelihood and Firth penalized estimates are both presented (Table S1). The subgroup analyses of first-attempt success, by contrast, were prespecified and registered.

A two-sided *p* value below 0.05 was taken as being significant. No outcome data were missing, so the prespecified rule to omit participants with missing data was never applied. All prespecified analyses were carried out in jamovi (version 2.7.38; The Jamovi Project, Sydney, Australia); the sensitivity analyses described below were performed in Python 3.11 with statsmodels 0.15.0.

Because the arms differed in age and in the distribution of insertion sites, the co-primary outcomes were re-estimated with adjustment. Age, a baseline characteristic, was adjusted for first; a second model added insertion site, which the operator chose after the allocation envelope had been opened and which is therefore a post-randomization variable. Adjusted median differences were obtained by median regression, which returns an estimate comparable with the unadjusted Hodges–Lehmann difference, with 95% confidence intervals from 5000 bootstrap resamples; linear models with heteroscedasticity-consistent (HC3) standard errors are reported alongside. First-attempt success was modeled by logistic regression with the same two covariate sets. These analyses were not prespecified (Table S2).

## 3. Results

### 3.1. Participant Flow and Baseline Characteristics

**Figure 1:**
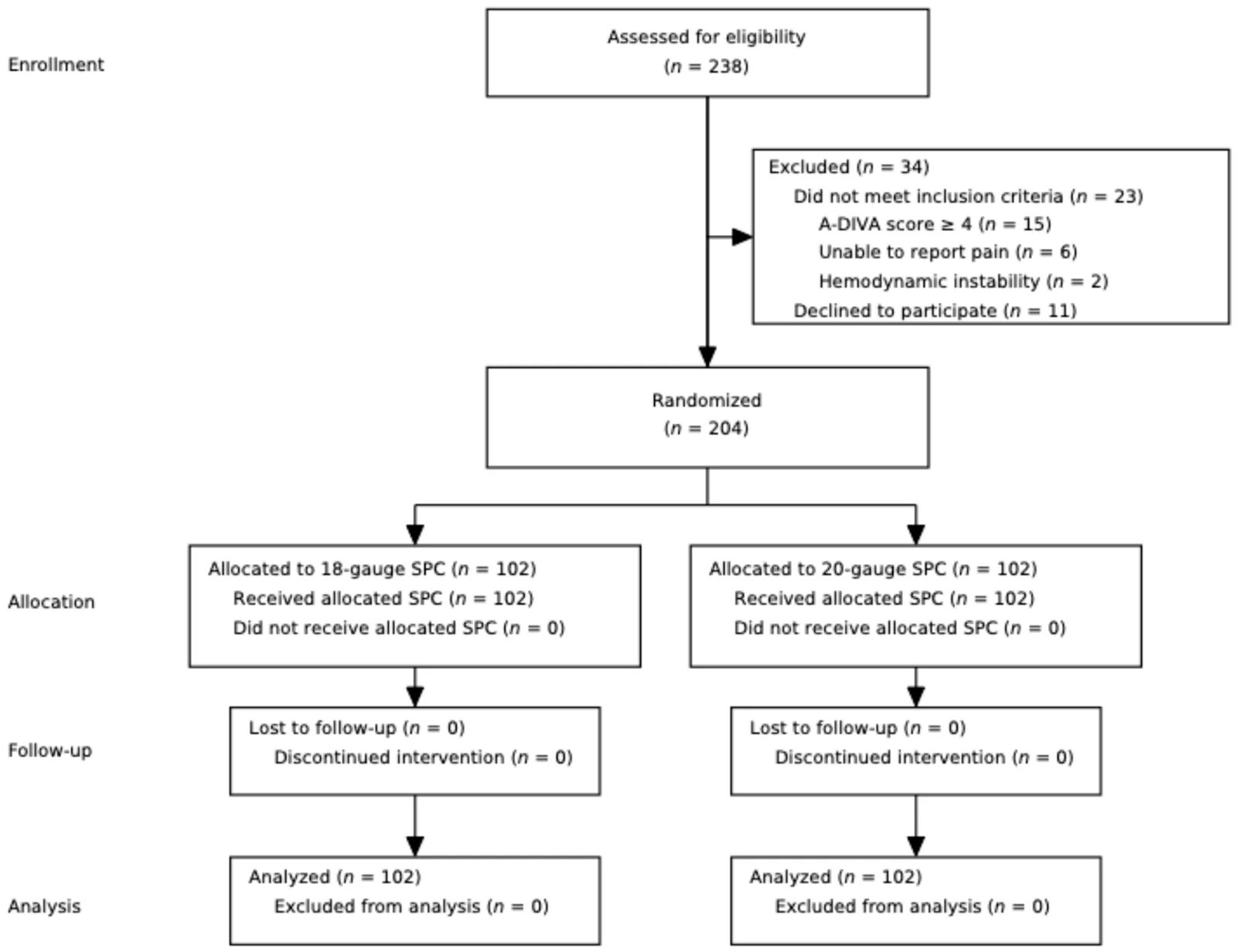
CONSORT flow diagram. Of 238 patients assessed for eligibility, 34 were excluded before randomization and 204 were allocated 1:1 to an 18-gauge or a 20-gauge short peripheral catheter. All received the allocated SPC, and no outcome data were missing, so the intention-to-treat and per-protocol populations were identical. SPC, short peripheral catheter.

In July 2026, 238 adults requiring peripheral intravenous access were screened. A total of 34 patients were excluded before randomization, 23 because they did not meet the inclusion criteria (A-DIVA score ≥ 4, *n* = 15; unable to report pain, *n* = 6; hemodynamic instability, *n* = 2) and 11 because they declined, and 204 were allocated to an 18-gauge (*n* = 102) or a 20-gauge (*n* = 102) SPC (Figure 1). All received the assigned SPC, and all 18 patients whose first attempt failed underwent the protocol-mandated second attempt with it. No patient was lost to follow-up, and no outcome data were missing, so the intention-to-treat and per-protocol populations were identical and gave identical results.

The median age was 48.5 years (IQR 34.0–65.2), 103 patients (50.5%) were men, and 167 (81.9%) were at low A-DIVA risk; the arms differed in age (median 44.0 vs. 52.0 years) but were otherwise comparable (Table 1). Insertion site and operator experience were determined after randomization by the unblinded operator and are reported separately (Table 2); the arms differed in the proportion cannulated in the forearm (21 vs. 12).

### 3.2. Primary Outcomes

Neither co-primary outcome was normally distributed (Shapiro–Wilk *p* < 0.01). Median pain after the first attempt was 32.5 mm with the 18-gauge SPC and 30.5 mm with the 20-gauge, a Hodges–Lehmann median difference of 1 mm [95% confidence interval (CI) −4 to 6; *p* = 0.65]; median procedural difficulty was 12.0 mm and 15.0 mm respectively, a difference of −1 mm (95% CI −4 to 1; *p* = 0.28) (Table 3).

### 3.3. First-Attempt Success

The first attempt succeeded in 92.2% of patients with the 18-gauge SPC and 90.2% with the 20-gauge (risk difference 2.0 percentage points, 95% CI −5.8 to 9.7; odds ratio 1.28, 95% CI 0.48–3.38; *p* = 0.62). Overall, 186 patients (91.2%) were cannulated on the first pass (Table 3). No insertion-related complications were recorded during the insertion attempt in either arm.

### 3.4. Subgroup Analyses

**Figure 2:**
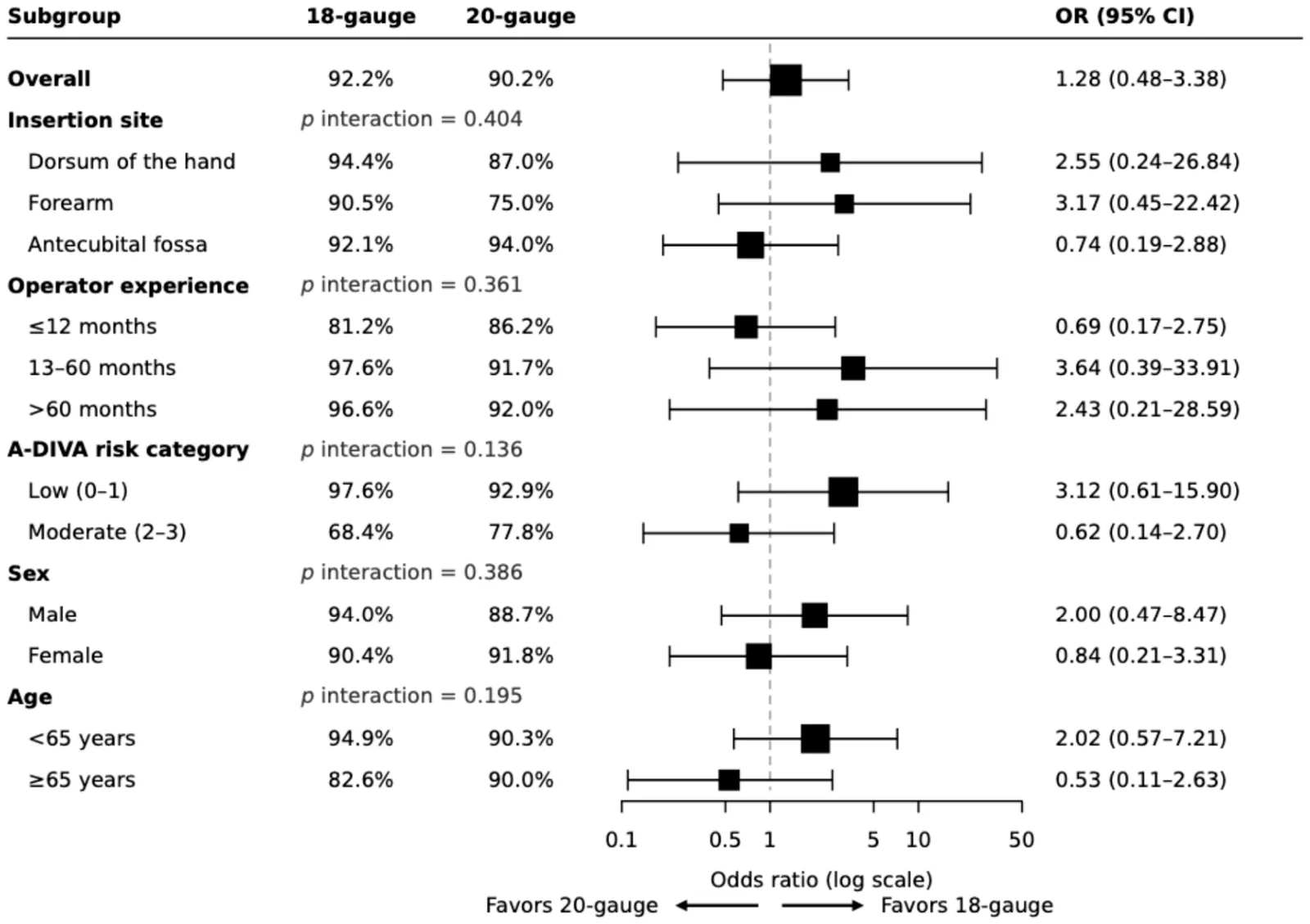
First-attempt success with 18-gauge versus 20-gauge short peripheral catheters, overall and by prespecified subgroup. Squares represent odds ratios for first-attempt success (18-gauge versus 20-gauge, with 20-gauge as the reference), sized in proportion to subgroup size; horizontal lines denote 95% confidence intervals; and the dashed vertical line indicates no difference between groups (odds ratio 1.0). Percentages are first-attempt success rates within each group. *p* values for interaction were derived from logistic regression models containing a treatment-by-subgroup interaction term. Subgroup analyses were prespecified but exploratory, were not powered to detect within-subgroup effects, and were not adjusted for multiple comparisons. A-DIVA, Adult Difficult Intravenous Access; CI, confidence interval; OR, odds ratio; SPC, short peripheral catheter.

First-attempt success was examined across the five prespecified subgroups, defined by insertion site, operator experience, A-DIVA risk category, patient age, and patient sex. Every confidence interval included an odds ratio of 1.0, and no interaction term approached significance (*p* = 0.136 to 0.404; individual values in Figure 2). With only 18 first-attempt failures in total, unevenly distributed across subgroups, these analyses had negligible power to detect interaction.

Post hoc estimates of pain and procedural difficulty within the insertion site, operator experience and A-DIVA subgroups all had confidence intervals that included zero (Table 4). The largest, at the dorsum of the hand (pain, +11 mm, 95% CI −4 to 26), was imprecise rather than small; none of these estimates are interpretable as an effect.

### 3.5. Post Hoc Multivariable Analysis of First-Attempt Success

In the post hoc multivariable model, SPC size remained unrelated to first-attempt success (adjusted OR 1.62, 95% CI 0.55 to 4.71; *p* = 0.38). A-DIVA risk category was associated with failure, success falling from 95.2% in low-risk patients to 73.0% in those at moderate risk (adjusted OR 0.13, 95% CI 0.04 to 0.37; *p* < 0.001). First-attempt success was lower among nurses with 12 months experience or less (83.6%) than in the two more experienced strata, in which it was identical (both 94.4%); neither adjusted estimate reached conventional significance. Firth penalized estimates were consistently attenuated towards the null and altered no conclusion (Table S1).

### 3.6. Sensitivity Analysis for Post-Randomization Imbalance

Adjustment did not alter either co-primary outcome. With adjustment for age, the median difference in pain was 0.2 mm (95% CI −5.8 to 10.4) and, in procedural difficulty, −3.0 mm (95% CI −6.6 to 2.7); adding insertion site yielded 2.2 mm (95% CI −6.3 to 10.1) and −3.0 mm (95% CI −7.1 to 2.1). Linear models with robust standard errors gave estimates of the same magnitude and direction. The upper limit of every interval for pain remained below the 13 mm minimum clinically important difference. First-attempt success was similarly unaffected (adjusted odds ratio 1.18, 95% CI 0.44 to 3.16 with age; 1.30, 95% CI 0.48 to 3.57 with age and insertion site). Full estimates are given in Table S2.

## 4. Discussion

In this randomized trial of 204 adults at low-to-moderate risk of difficult venous access, we detected no difference between the two sizes in any of the three outcomes. Pain after the first attempt differed by 1 mm (95% CI −4 to 6) and procedural difficulty differed by −1 mm (95% CI −4 to 1) on a 100 mm scale, and the first attempt succeeded in 92.2% of patients with the 18-gauge SPC and 90.2% with the 20-gauge (odds ratio 1.28, 95% CI 0.48 to 3.38). The trial was powered to detect a 13 mm difference in pain—the minimum change considered clinically meaningful by patients [14,15,16]—but found no statistically significant difference between the groups. Both limits of the observed interval lie well within that margin, so the data are compatible only with differences smaller than the trial set out to detect. The findings are consistent with the absence of a clinically important difference, but formal equivalence between 18-gauge and 20-gauge SPCs was not tested: the trial was designed to detect superiority, and no equivalence margin was specified in advance.

Two earlier studies have addressed this question directly, and both reported null results. Among 1063 surgical patients, van Loon and colleagues found that SPC size had no independent association with insertion pain once sex, ASA classification, A-DIVA risk, puncture site, and the success of the attempt were accounted for, and the 18-gauge SPC recorded the lowest mean score [10]. The SPICED trial randomized 178 emergency patients to an 18-gauge or a 20-gauge SPC and found no difference in pain, in procedural difficulty or in first-attempt success [8]. Neither study used a validated measure of access difficulty to define eligibility, nor judged its result against a threshold of clinical importance set beforehand. Our trial adds both and reaches the same conclusion. Its contribution is therefore confirmatory rather than novel: two randomized trials, conducted in different health systems and under different eligibility criteria, now concur that gauge does not determine insertion pain or procedural difficulty, and the present trial further demonstrates that any difference in pain lies below the threshold accepted as clinically important.

The findings for procedural difficulty paralleled those for pain. Both SPCs were rated as easy to place, with medians of 12.0 mm and 15.0 mm on a 100 mm scale, which offers no support for the common belief that a wider SPC is harder to insert. Insertion site appears to matter more than gauge. Puncture site was an independent predictor of pain in van Loon’s model, while SPC size was not [10], and in the randomized comparison reported by Kaplan and colleagues, insertion in the forearm was less painful than insertion in the dorsum of the hand [17]. Our own estimates by site were post hoc and too imprecise to address this (Table 4). The belief that gauge governs difficulty may nonetheless be self-sustaining: in the ‘’Peripheral Intravenous Catheter Management in Childbirth’’ (PICMIC) cohort, the gauge a clinician selected was determined by their confidence in placing it [18].

First-attempt success was 91.2% overall, above the 73% and 86% reported in unselected emergency department cohorts [5,6] and the 81% of a multicenter hospital-wide cohort [12]. Restricting enrollment to an A-DIVA score of 0 to 3 excluded the patients in whom failure concentrates, which is the likely explanation. Within this population, success was unrelated to SPC size after adjustment (adjusted OR 1.62, 95% CI 0.55 to 4.71) but was related to access risk, falling from 95.2% at low risk to 73.0% at moderate risk (adjusted OR 0.13, 95% CI 0.04 to 0.37), a gradient also seen in the derivation cohort [2]. Success was lower among nurses with 12 months of experience or less, although these estimates did not reach conventional significance. The direction agrees with published data: greater cumulative insertion experience predicted first-time success in two emergency department cohorts [5,6], and among older emergency department patients, nurses with less than two years of experience had a more than threefold higher rate of first-attempt failure [19]. This model was post hoc, contained only 18 events and compares groups that were not randomized, so it describes association rather than effect. The pattern suggests that first-attempt success is governed by the operator and by the patient’s baseline access risk rather than by the SPC.

The choice of gauge, however, is not settled by pain and insertion difficulty alone. Contemporary vascular access guidance approaches the decision from the vessel rather than from the device, holding that the caliber of the catheter should be matched to the caliber of the vein that will receive it and to the therapy the patient is expected to need, so that sufficient flow is preserved around the catheter within the lumen. The recently published SISPeC bundle, developed by GAVeCeLT and IVAS, places this assessment at the beginning of the insertion sequence: both arms are examined with and without a tourniquet before a vein is selected, preference is given to veins of the forearm or to the cephalic vein at the upper arm, and areas of flexion such as the wrist and the antecubital fossa are avoided [20]. A catheter that is large in relation to the vein it occupies, or one sited where the limb bends, is held to be at greater risk of infiltration, phlebitis, thrombosis, and premature failure, and both catheter characteristics and insertion site were associated with failure in a secondary analysis of 11,830 catheters [21]. Our own insertions were concentrated in the antecubital fossa, which accounted for 63.7% of first attempts, a distribution that reflects prevailing practice in our department rather than the site selection this bundle recommends. The present findings therefore remove one consideration from the decision—that a wider SPC will hurt more or prove harder to place—without addressing those that govern how long the device remains serviceable once in place. Gauge selection remains a judgment about the vein and the anticipated therapy, and this trial narrows that judgment rather than resolving it.

For practice, this means that in adults at low-to-moderate risk of difficult access, the pain and difficulty expected from a wider SPC carry less weight in the decision than is commonly assumed. The choice can follow the therapy the patient is likely to need: the rate of fluid resuscitation, the administration of blood products, or the injection rate required for contrast-enhanced imaging. Where a wider SPC may be needed, the present data give no reason to expect that placing it will cost the patient comfort or the operator ease of insertion, and the selection of a narrower SPC with the intention of reducing discomfort is not supported by these findings. In patients at higher access risk, whom we did not study, the intervention that improves first-attempt success is ultrasound guidance rather than a change of gauge; it raised first-attempt success by a risk ratio of 1.80 (95% CI 1.18 to 2.73) across eight emergency department trials [22]. Efforts to make cannulation less unpleasant are therefore better directed at operator training and at identifying difficult access before the first attempt than at the size of the SPC.

### Limitations

Several limitations qualify these findings, and the first concerns the patients to whom they apply. Enrollment was restricted to an A-DIVA score of 0 to 3, and within that range, the sample was weighted towards the easier end: 167 of 204 patients (81.9%) were at low risk, leaving 37 at moderate risk. Patients scoring 4 or 5, and those who were hemodynamically unstable, were excluded altogether. The trial therefore speaks to patients whose veins are comparatively easy to access, and not to the two groups in whom the choice of gauge is most often debated—patients with difficult access and those undergoing active resuscitation. The gradient within the trial illustrates the point: first-attempt success fell from 95.2% at low risk to 73.0% at moderate risk, and it is the moderate stratum, with 37 patients, that lies closest to the population in question. These findings should not be extrapolated beyond the population studied. The trial was also conducted at a single center with SPCs from a single manufacturer, and both the case mix and the skill profile of the nursing staff may differ elsewhere.

The operator’s involvement extended beyond performing the procedure, and this bears on two measurements. The A-DIVA score was assigned by the nurse who then performed the cannulation, and an operator who expects a difficult attempt may approach it differently; anticipated difficulty carried the largest odds of first-attempt failure among older emergency department patients [19], so the risk strata used here are not independent of the operator. Procedural difficulty is the outcome most exposed to bias: unlike pain, which patients scored under blinding, it was rated by the operator. The SPCs are color-coded, so neither the operator nor any bedside observer could be blinded, and the operator who learned the allocation also chose the vein, performed the puncture and rated the difficulty. Allocation awareness could therefore have acted at two points. It could have acted on the rating itself, in either direction, if an operator expected the wider catheter to be harder to place. It could also have acted before the puncture: the envelope was opened before the site was selected, so an operator anticipating difficulty could have sought a larger or more accessible vein for that arm, lowering the recorded difficulty without leaving any trace in the rating. The imbalance in forearm insertions (21 vs. 12) is compatible with such selection, though chance remains an equally plausible explanation. An unbiased estimate of this outcome would require an assessor blinded to the hub color, whether through video review or through objective proxies such as the number of skin punctures or the time taken to obtain access. No minimum important difference has been validated for a procedural difficulty scale, so those scores cannot be judged against an external benchmark as the pain scores can.

The trial was designed to detect a difference between the two sizes rather than to establish that they are equivalent, and no equivalence margin was set in advance. SPCs were observed only during the insertion attempt and were not followed after the emergency department visit, so dwell time, phlebitis, infiltration and catheter failure were not assessed, and these have been associated with catheter characteristics and insertion site in large observational cohorts [21]. The post hoc analyses reported in Table 4 and Table S1 remain exploratory. Patients allocated to the 18-gauge SPC were somewhat younger and were more often cannulated in the forearm. Adjustment for age, and for age together with insertion site, left every estimate unchanged (Table S2). Such adjustment cannot, however, address the concern raised above: insertion site was chosen after the allocation was known, so conditioning on it does not restore the protection that randomization affords.

## 5. Conclusions

In adults at low-to-moderate risk of difficult intravenous access, we did not detect a difference between 18-gauge and 20-gauge SPCs in insertion pain, in procedural difficulty, or in first-attempt success. For pain, the confidence interval lay well within the accepted threshold of clinical importance, so the data leave little room for a difference that patients would notice. The findings are therefore consistent with the absence of a clinically important difference, but formal equivalence was not tested, and no equivalence margin was set in advance. In this population, SPC size can therefore be chosen based on the therapy the patient is likely to need rather than on the discomfort the insertion is expected to cause. Because four in five participants were at low risk of difficult access, the finding should not be carried across to patients with difficult access, in whom gauge selection is most often debated; that population, and outcomes arising after the insertion attempt, require separate study.

## Figures and Tables

**Table 1:** Baseline characteristics, overall and by allocated SPC size.

Characteristics	All *n* = 204	SPC Size	
		18-Gauge, *n* = 102	20-Gauge, *n* = 102
Age (years)	48.5 (34.0–65.2)	44.0 (32.2–62.7)	52.0 (36.2–68.7)
Sex			
Male	103 (50.5)	50 (49.0)	53 (52.0)
Female	101 (49.5)	52 (51.0)	49 (48.0)
A-DIVA score			
0–1 (low)	167 (81.9)	83 (81.4)	84 (82.4)
2–3 (moderate)	37 (18.1)	19 (18.6)	18 (17.6)

Data are median (IQR, 25th–75th percentile) or *n* (%). A-DIVA, Adult Difficult Intravenous Access; IQR, interquartile range; SPC, short peripheral catheter.

**Table 2:** Procedural characteristics, overall and by allocated SPC size.

Characteristics	All *n* = 204	SPC Size	
		18-Gauge, *n* = 102	20-Gauge, *n* = 102
Insertion site			
Dorsum of hand	41 (20.1)	18 (17.6)	23 (22.5)
Forearm	33 (16.2)	21 (20.6)	12 (11.8)
Cubital fossa	130 (63.7)	63 (61.8)	67 (65.7)
Operator experience (months)			
≤12	61 (29.9)	32 (31.4)	29 (28.4)
13–60	89 (43.6)	41 (40.2)	48 (47.1)
>60	54 (26.5)	29 (28.4)	25 (24.6)

Data are *n* (%). Insertion site and operator experience were determined after randomization by the unblinded operator, who selected the vein and site once the allocation envelope had been opened; they are therefore procedural rather than baseline characteristics; SPC, short peripheral catheter.

**Table 3:** Primary and secondary outcomes: insertion pain, procedural difficulty, and number of cannulation attempts with 18-gauge versus 20-gauge short peripheral catheters.

	All *n* = 204	SPC Size		OR (95% CI) or Median Difference (95% CI)
		18-Gauge, *n* = 102	20-Gauge, *n* = 102	
Pain VAS, first attempt	32.0 (17.7–46.0)	32.5 (18.0–48.0)	30.5 (17.2–44.0)	1 (−4 to 6)
Difficulty VAS, first attempt	12.5 (8.0–21.5)	12.0 (7.2–20.0)	15.0 (8.0–23.5)	−1 (−4 to 1)
Number of attempts				
1	186 (91.2)	94 (92.2)	92 (90.2)	1.28 (0.48–3.38)
2	15 (7.3)	6 (5.9)	9 (8.8)	*
≥3	3 (1.5)	2 (2.0)	1 (1.0)	*

Data are median (IQR, 25th–75th percentile) or *n* (%). * Could not be calculated due to small group numbers. Median differences are Hodges–Lehmann estimates for 18-gauge minus 20-gauge; a positive value indicates higher pain or difficulty with the 18-gauge SPC. The odds ratio compares first-attempt success (18-gauge versus 20-gauge). CI, confidence interval; IQR, interquartile range; SPC, short peripheral catheter; OR, odds ratio; VAS, visual analog scale.

**Table 4:** Subgroup analysis of pain and procedural difficulty VAS scores by allocated SPC size.

	*n* (18/20 Gauge)	Pain VAS			Difficulty VAS		
		18-Gauge	20-Gauge	Median Difference (95% CI)	18-Gauge	20-Gauge	Median Difference (95% CI)
Insertion site							
Dorsum of hand	18/23	48.5 (26.7–59.2)	32.0 (18.0–45.0)	11 (−4 to 26)	12.0 (10.0–17.0)	12.0 (7.5–21.5)	0.0 (−6 to 6)
Forearm	21/12	41.0 (31.0–51.0)	30.0 (17.7–41.7)	7 (−4 to 19)	17.0 (12.0–23.0)	15.5 (8.0–26.7)	1.5 (−7 to 9)
Cubital fossa	63/67	25.0 (15.0–40.5)	30.0 (17.5–43.0)	−3 (−9 to 3)	11.0 (7.0–18.0)	15.0 (8.5–24.5)	−3 (−6 to 0)
Operator experience (months)							
≤12	32/29	38.0 (26.5–46.0)	34.0 (24.0–39.0)	4 (−3 to 12)	19.0 (12.0–35.2)	20.0 (10.0–28.0)	3 (−4 to 10)
13–60	41/48	34.0 (15.0–48.0)	28.5 (16.0–42.5)	3 (−4 to 11)	10.0 (7.0–18.0)	10.5 (7.0–20.2)	−1 (−4 to 2)
>60	29/25	22.0 (11.0–47.0)	28.0 (17.0–49.0)	−6 (−18 to 5)	10.0 (7.0–15.0)	16.0 (8.0–22.0)	−4 (−10 to 0)
A-DIVA score							
0–1 (low)	83/84	28.0 (16.5–45.5)	26.0 (16.7–41.0)	2 (−3 to 7)	11.0 (7.0–17.5)	11.0 (8.0–21.0)	−1 (−4 to 1)
2–3 (moderate)	19/18	46.0 (30.0–56.0)	45.0 (35.0–64.2)	−3.5 (−20 to 11)	26.0 (13.0–46.0)	23.0 (15.0–35.5)	2 (−10 to 15)

Data are median (IQR, 25th–75th percentile). These subgroup analyses were post hoc and exploratory. A-DIVA, Adult Difficult Intravenous Access; CI, confidence interval; IQR, interquartile range; VAS, visual analog scale.

## Data Availability

The raw data supporting the conclusions of this article will be made available by the authors on request.

## Supplementary Materials

The following supporting information can be downloaded at: https://www.mdpi.com/article/10.3390/medicina62091747/s1. Table S1: Post hoc multivariable analysis of first-attempt success; Table S2: Sensitivity analysis of the trial outcomes with adjustment for age and insertion site.
