# Supplementary material for: A Comparison of 18-Gauge and 20-Gauge Short Peripheral Catheters in Terms of Pain and Procedural Difficulty: A Randomized Controlled Trial in the Emergency Department
Source: Medicina (Kaunas). 2026 Sep 10;62(9):1747. doi: 10.3390/medicina62091747 (PMC13609332; doi:10.3390/medicina62091747)
Supplement: Supplementary file 1 [file medicina-62-01747-s001.zip › medicina-4512701-supplementary.pdf]

Supplementary Materials

A Comparison of 18-Gauge and 20-Gauge Short Peripheral Catheters in Terms of Pain and Procedural Difficulty: A Randomized Controlled Trial in the Emergency Department

Table S1. Post hoc multivariable analysis of first-attempt success.

Variable	First-attempt success, *n/N* (%)	Maximum likelihood OR (95% CI)	*p*	Firth OR (95% CI)	Firth *p*
		SPC size			
20-gauge (reference)	92/102 (90.2)	1.00	—	1.00	—
18-gauge	94/102 (92.2)	1.62 (0.55–4.71)	0.380	1.55 (0.56–4.52)	0.397
		A-DIVA risk category			
Low, score 0–1 (reference)	159/167 (95.2)	1.00	—	1.00	—
Moderate, score 2–3	27/37 (73.0)	0.13 (0.04–0.37)	<0.001	0.14 (0.05–0.38)	<0.001
		Operator experience			
≤12 months (reference)	51/61 (83.6)	1.00	—	1.00	—
13–60 months	84/89 (94.4)	3.21 (0.98–10.60)	0.055	2.98 (0.98–10.00)	0.054
>60 months	51/54 (94.4)	4.07 (0.96–17.33)	0.057	3.53 (0.99–15.96)	0.052

n, first-attempt successes; *N*, insertions in that category; percentage = n/N × 100. Odds ratios are mutually adjusted for the other variables shown; values above 1.00 indicate higher odds of first-attempt success than the reference category. Maximum-likelihood estimates are shown with Wald 95% confidence intervals; Firth penalized estimates are shown with profile-likelihood 95% confidence intervals and penalized likelihood-ratio *p* values. Firth penalization was applied, and the model was restricted to three variables, because only 18 first-attempt failures occurred, giving 4.5 events per estimated coefficient, at which maximum-likelihood estimates are biased away from the null. Associations for A-DIVA risk and operator experience are not based on randomized comparisons. This analysis was not prespecified and is exploratory. A-DIVA, Adult Difficult Intravenous Access; CI, confidence interval; OR, odds ratio; SPC, short peripheral catheter.

Table S2. Sensitivity analysis of the trial outcomes with adjustment for age and insertion site.

Outcome and model	Estimate (95% CI)	*p*
Pain VAS, median difference (mm)		
Unadjusted	1.0 (−4.0 to 6.0)	0.65
Model 1: age	0.2 (−5.8 to 10.4)	0.88
Model 2: age + insertion site	2.2 (−6.3 to 10.1)	0.64
Pain VAS, mean difference (mm)		
Model 1: age	1.4 (−3.9 to 6.7)	0.60
Model 2: age + insertion site	1.3 (−4.0 to 6.5)	0.64
Procedural difficulty VAS, median difference (mm)		
Unadjusted	−1.0 (−4.0 to 1.0)	0.28
Model 1: age	−3.0 (−6.6 to 2.7)	0.46
Model 2: age + insertion site	−3.0 (−7.1 to 2.1)	0.41
Procedural difficulty VAS, mean difference (mm)		
Model 1: age	−1.0 (−4.7 to 2.7)	0.59
Model 2: age + insertion site	−1.3 (−5.1 to 2.5)	0.51
First-attempt success, odds ratio		
Unadjusted	1.28 (0.48 to 3.38)	0.62
Model 1: age	1.18 (0.44 to 3.16)	0.74
Model 2: age + insertion site	1.30 (0.48 to 3.57)	0.61

Estimates compare the 18-gauge with the 20-gauge SPC: a positive difference indicates a higher score, and an odds ratio above 1.00 higher odds of first-attempt success, with the 18-gauge SPC. Model 1 adjusts for age, a baseline characteristic. Model 2 additionally adjusts for insertion site, which the operator selected after the allocation envelope had been opened and which is therefore a post-randomization variable; conditioning on it departs from the intention-to-treat principle, and these estimates are interpreted with that caveat. Median differences are from median (quantile) regression with percentile 95% confidence intervals from 5000 bootstrap resamples; the unadjusted median differences are Hodges–Lehmann estimates with bootstrap confidence intervals. Mean differences are from linear models with heteroscedasticity-consistent (HC3) standard errors, and odds ratios from logistic regression with Wald confidence intervals. Insertion site was modelled as a three-level categorical variable with the cubital fossa as the reference. These analyses were not prespecified and are exploratory. CI, confidence interval; SPC, short peripheral catheter; VAS, visual analog scale.
